# Memristor Based Binary Convolutional Neural Network Architecture With Configurable Neurons

**DOI:** 10.3389/fnins.2021.639526

**Published:** 2021-03-26

**Authors:** Lixing Huang, Jietao Diao, Hongshan Nie, Wei Wang, Zhiwei Li, Qingjiang Li, Haijun Liu

**Affiliations:** ^1^College of Electronic Science and Technology, National University of Defense Technology, Changsha, China; ^2^College of Electrical and Information Engineering, Hunan University, Changsha, China

**Keywords:** memristor, binarized neural networks, convolutional neural networks, device defects effect, configurable neuron, neuromorphic computing

## Abstract

The memristor-based convolutional neural network (CNN) gives full play to the advantages of memristive devices, such as low power consumption, high integration density, and strong network recognition capability. Consequently, it is very suitable for building a wearable embedded application system and has broad application prospects in image classification, speech recognition, and other fields. However, limited by the manufacturing process of memristive devices, high-precision weight devices are currently difficult to be applied in large-scale. In the same time, high-precision neuron activation function also further increases the complexity of network hardware implementation. In response to this, this paper proposes a configurable full-binary convolutional neural network (CFB-CNN) architecture, whose inputs, weights, and neurons are all binary values. The neurons are proportionally configured to two modes for different non-ideal situations. The architecture performance is verified based on the MNIST data set, and the influence of device yield and resistance fluctuations under different neuron configurations on network performance is also analyzed. The results show that the recognition accuracy of the 2-layer network is about 98.2%. When the yield rate is about 64% and the hidden neuron mode is configured as −1 and +1, namely ±1 MD, the CFB-CNN architecture achieves about 91.28% recognition accuracy. Whereas the resistance variation is about 26% and the hidden neuron mode configuration is 0 and 1, namely 01 MD, the CFB-CNN architecture gains about 93.43% recognition accuracy. Furthermore, memristors have been demonstrated as one of the most promising devices in neuromorphic computing for its synaptic plasticity. Therefore, the CFB-CNN architecture based on memristor is SNN-compatible, which is verified using the number of pulses to encode pixel values in this paper.

## Introduction

In recent years, the neuromorphic hardware inspired by human brain has aroused researchers’ attention (Mead, 1990). Compared with traditional hardware, including FPGA, GPU, and CPU (Boybat et al., 2018), brain-inspired neuromorphic computing systems is more efficient to implement deep neural networks for their less power consumption and quicker computing speed. Although the neuromorphic computing system based on complementary-metal-oxide-semiconductor (CMOS) has achieved a great progress, constrained by limited area in portable devices, there remains huge challenge to implement a brain-level neuromorphic system based on the standard CMOS technology (Wang et al., 2018).

Memristor has the advantages of low consumption power, nanoscale size, non-volatile storage, and multi-value tune-ability (Hu et al., 2014). And the memristor array (Strukov et al., 2008) can be used to execute vector-matrix multiplication (VMM) in one step naturally, therefore, memristor has been regarded as one of the most potential devices for implementing neuromorphic computing such as convolutional neural networks (CNNs) (Yao et al., 2020), spiking neural networks (SNNs) (Midya et al., 2019; Nikiruy et al., 2019), and multi-layer perceptron (MLP) (Pham et al., 2018). Among them, the research on CNNs (Yakopcic et al., 2016, 2017; Sun et al., 2018) is of great significance, as it has present excellent performance in object recognition (Krizhevsky et al., 2017) and detection (Girshick, 2015).

Yakopcic et al. (2015; 2016; 2017) proposed a neuromorphic circuit using memristor to mitigate the problem caused by existing parallel structure of the crossbar, and then used this circuit to implement a CNN algorithm based on *ex-situ* training. Additionally, the first memristor based circuits that can completely parallelize the recognition of CNN have been presented by them. In order to tackle the problem of specific object recognition based on memristor array circuits, Sun et al. (2019a) proposed Full-Parallel Convolutional Neural Networks (FP-CNN) for generating multiple output feature maps in one step processing cycle, and the three-layer architecture of the FP-CNN achieves 99% classification accuracy. Zeng et al. (2018) proposed a new memristor architecture for image convolution calculations, which outputs just one element at a time, and they proposed an algorithm for convolution on an image for this architecture so that a complete feature map can be generated.

In many cases, the research concluded above considered the memristor as the device with multi-level or precise memristance. Constrained by the manufacturing technology, the devices with multi-level memristance are not stable or easily and successfully prepared on a large scale, whereas the memristor devices with binary value (namely high resistance state HRS and low resistance states LRS) are basically available. Besides, the current memristor based CNN architecture usually has just one type of activation function, which is fulfilled in high precision and failed to be configurable automatically, exploited in the hidden layer. Therefore, from the perspective of reducing overhead and complexity of hardware, we propose a configurable full-binary convolutional neural network (CFB-CNN) architecture. Our contributions can be summarized as following:

• At the algorithm-level, we propose a fully binarized network architecture with configurable neurons. This architecture also achieves good performance under the condition that the memristor array has inherent defects like yield rates and memristance variation.
• We compare our CFB-CNN architecture with other state-of-the-art CNN architectures whose inputs, weights, and neurons are high precision in terms of classification accuracy on MNIST dataset. In addition, the hardware overheads of the CFB-CNN architecture are presented. Through experiments, we found that, at the cost of less hardware resource consumption, the recognition rate of the CFB-CNN architecture on the MNIST dataset is just reduced by 0.8%.
• We also verify the feasibility of our architecture on FASHION-MNIST dataset. From our experiments, the classification accuracy on this dataset achieves to 86.97%, which shows 2.71% decline compared with basic computation unit (Sun et al., 2019b), a conventional CNN with full precision.
• What’s more, in order to prove that the memristor-based CFB-CNN architecture is compatible with SNN, we use the number of pulses to encode the pixel value of the images in the MNIST data set for conducting related simulation experiments. And the results show that the memristor-based CFB-CNN architecture is well compatible with SNN.
• Last but not least, we give the scheme of implementing configurable neurons at the circuit level, and then investigate the impact caused by the hidden neuron output fluctuation on the recognition rate of the CFB-CNN architecture.

The rest of this paper is organized as following. Section II details the architecture of CFB-CNN. Section III elaborates the process of implementing CFB-CNN architecture on memristor array and analyses the defects influences to the network. Section IV concludes the paper.

## Architecture of CFB-CNN

### Convolutional Neural Network

**FIGURE 1:**
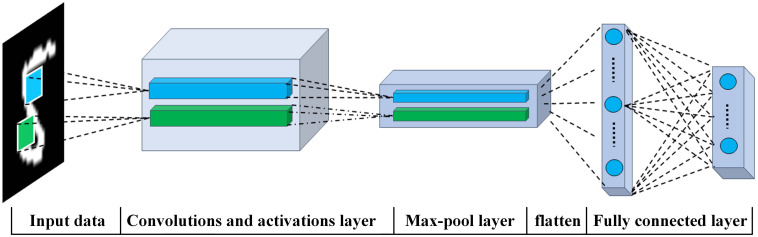
A typical architecture of convolutional neural network.

A typical architecture of CNN applied in most image classification tasks can be depicted as Figure 1.

As shown in Figure 1, a classic CNN is made up of two modules: the first one is served as a feature extractor, which is equipped with convolution and pooling layer, and its output feature maps should be sent to the second module, namely the fully connected layer, which is functioned as a classifier to determine which category to be the winner through calculating the probability of each category.

### The Topology of CFB-CNN

The architecture of the CFB-CNN builds on our previous FP-CNN (Sun et al., 2019a), whose architecture is the same as the typical CNN architecture. In our previous design, the pooling layer consumes a certain number of op-amps, increasing the complexity of hardware implemented for CNN. Meanwhile, the weights and neurons in FP-CNN are high precision requiring memristor equipped with multilevel resistance state and relatively complex circuits for activation function, which is challenging for implementing memristor based CNN. Therefore, for better realization of memristor CNN, we have investigated the all binarized CNN without pooling layer, while neurons in hidden layer are configurable.

**FIGURE 2:**
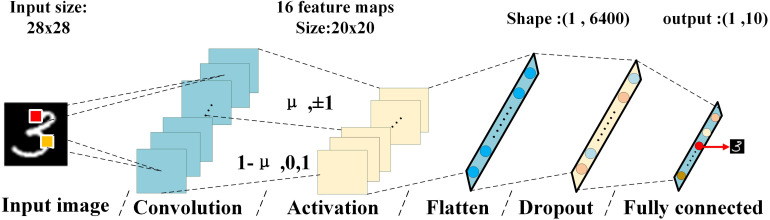
Diagram of the CFB-CNN architecture, the input of the fully connected layer is the flattening 16× feature maps, the top labels give the information about input image size, kernel size, number of kernels, and number of neurons in the hidden layer.

In this work, the CFB-CNN architecture is the simplest version of the CNN architecture described in Figure 1, consisting in just one feature extractor module without pooling layer and a classifier that just with one fully connected layer. Figure 2 shows the architecture of CFB-CNN.

**FIGURE 3:**
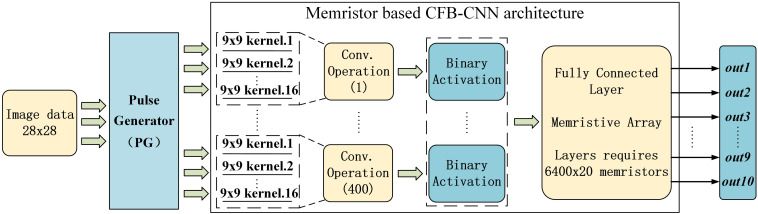
Data flow of CFB-CNN architecture.

In the convolutional layer, there are sixteen 9 × 9 kernels. Since the values of the input image and the filter are binarized to 0, 1, and +1, −1, each kernel scans across the input image with a stride of 1 × 1 and performs bitwise vector matrix multiplication. These operations will generate sixteen output feature maps with size being 20 × 20. And the activation layer is divided into two proportionally adjustable parts. Specifically, the first part binarizes the pixel of the feature maps into +1 or −1 using the function of *binary tanh*, and the other binarizes the maps into 0 or 1. And the parameter of the proportion μ is used to determine how many feature maps are entered into the first part. Especially, when all feature maps are binarized to ±1, the CFB-CNN architecture is referred as ±1 MD, on the contrary, it would be referred to 01 MD when all feature maps are binarized to 0 or 1. In the training process, the percentage of activation with ±1 is μ, while that with 0 or 1 is 1-μ. The value of μ remains constant during training, and the training method is consistent with the usual training method of binary CNN. During the testing process, the weights after well trained and the value of μ that has been set during training are used for completing classification tasks. Figure 3 elaborates the data flow of the CFB-CNN architecture and gives the detail information about hardware overheads for fully connected layer.

At first, the input images are binarized to 0 and 1, then all the binarized pixels would be converted to a 1 × 784 pulses voltage output vector with amplitude information by the Pulse Generator (PG), and the vector is taken as the input dataflow of the memristor based CFB-CNN architecture. The pulses enter the memristor convolution kernel for performing parallel convolution operations. The output of the convolution operations is activated by the function of *binary tanh* and *binary sigmoid*. After that, the activation results are transferred to the fully connected layer as inputs. The number of outputs in the fully connected layer is the same as that of categories, and the pulse signal with the largest amplitude in output denotes the final classification result of the input image.

### Training Method of CFB-CNN

By training and testing the CFB-CNN architecture on the training set of MNIST, the well-trained weights and baseline are obtained for verifying the implementation feasibility. At first, the model refereed as ±1 MD is trained firstly. During the training procedure, the MNIST dataset has been separated into three parts, namely training set, validation set and testing set, each of them contains 55000, 5000, and 10000 images, respectively. 100 epochs are set for training, and each epoch complete iterations on 550 batches with batch size being 100. The method of exponentially decaying learning rate is adopted, and the initial learning rate is 0.01. Additionally, the moving average model is used, and the moving average decay rate is 0.99. The input images are binarized to 0,1 according to Eq. 1.

(1) $$f\left(x\right)=\left\{ \begin{array}{c} 0,x\leq 0.5\\ +1,x>0.5 \end{array}\right.$$

And the neurons are binarized according to Eq. 2, specifically, neurons determined by parameter μ are binarized to ±1 using the expression on the left, while the rest neurons are binarized to 0 or 1 using the expression on the right. And the weights are also binarized to ±1 using the left expression. It should be noticed that the network is trained with 1-bit precision in the forward computation but 32-bit floating point precision in back propagation (Courbariaux and Bengio, 2016), to be more specific, the gradient of tanh function is used to approximate the gradient of binary activation function.

(2) $$f\left(x\right)=\left\{ \begin{array}{c} -1,x\leq 0\\ +1,x>0 \end{array}\right.\text{or}f\left(x\right)=\left\{ \begin{array}{c} 0,x\leq 0\\ +1,x>0 \end{array}\right.$$

After the architecture of ±1 MD is well-trained, the rest 10000 images are used to test its performance of recognition. The results show that the CFB-CNN architecture behaves well with 98.2% classification accuracy. Then the mode of neurons is modified to be 0,1 and the network, namely 01 MD, is retrained under the same circumstance. For testing the same 10000 images, the classification accuracy is 98.22%.

Furthermore, for the reason that the memristor conductance (synaptic weight) is able to be modulated by the number of pulses, which means that the input of the memristor based CFB-CNN architecture can be applied in the form of pulses, we also test the performance of memristor based CFB-CNN architecture by applying a number of pulses determined by the pixel value of the image. Here, in order to facilitate the encoding of the image pixels in the form of the number of pulses, the image pixels are compressed into 4 bits for representation. For the model of 01 MD, the testing images compressed into 4 bits are directly input into the CFB-CNN architecture (4-bit-01 MD) which is well-trained according to Eqs 1, 2, and the recognition rate is 97.73%. While for the model of ±1 MD, the training dataset of MNIST are compressed into 4 bits for retraining the CFB-CNN architecture (4-bit-±1 MD). After the 4-bit-±1 MD is well-trained, the rest 10000 images are also compressed as the same way and the recognition rate of the ±1 MD is 98.37%. And the good recognition results show that the input of the network architecture is able to be encoded by number of identical spikes, that is to say, the CFB-CNN architecture is SNN-compatible.

Additionally, the CFB-CNN architecture is also tested on the FASHION-MNIST. With other things being equal, the number of the kernels and the kernel size to is 32 and 7 × 7, then the hidden neurons are binarized to 0 or 1 according to Eq. 2, while the input images are binarized to ±1 according to Eq. 3.

(3) $$f\left(x\right)=\left\{ \begin{array}{c} -1,x\leq 0\\ +1,x>0 \end{array}\right.$$

The classification accuracy on FASHION-MNIST is 86.97% which is 2.71% lower than that of the work (Sun et al., 2019a).

### Hardware Implementation of CFB-CNN

As mentioned above, the CFB-CNN architecture is the simplest one without pooling-layer, compared with a classical three-layer CNN architecture (Sun et al., 2019a), viz., convolution layer, pooling layer and fully connected layer. In realizing hardware CFB-CNN architecture, memristors are used to store the weighs and integrated into an array with one memristor at each cross point to execute VMM operation existed in the CFB-CNN architecture. The inputs to the row lines of the array are in the form of pulse voltage, and the results obtained from the column are in the form of current according to the Kirchhoff’s law. The weights in the CFB-CNN architecture are programmed to the conductance value of the memristors. Because all weights in the CFB-CNN architecture must be binarized to +1 or −1 and the memristance cannot be negative, two memristors are used to represent one weight. When the value of the weight is +1, the two memristors would be programmed to (G_LRS_, G_HRS_), otherwise, they would be programmed to (G_HRS_, G_LRS_). Eq. 4 reflects the relationship between the weights in CFB-CNN and conductance stored in memristor array.

(4) $$y=kx=\left(G_{LRS}-G_{HRS}\right)w_{i}$$

where *w*_*i*_ represents the weights in CFB-CNN, and the parameter *y* is the target conductance mapped by the corresponding weights.

**FIGURE 4:**
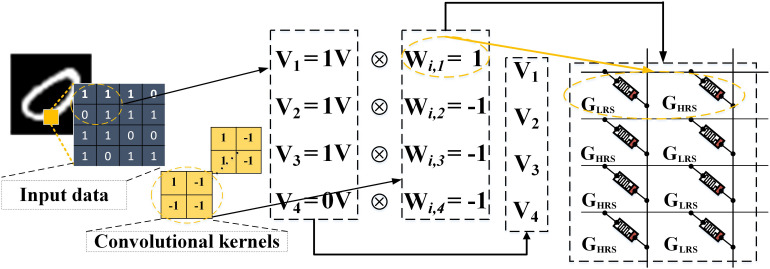
The demo about how to execute a convolution operator by using memristor crossbar. The size of the convolutional kernel is 2 × 2. The inputs sent to the array are in the form of DC voltage, and each weight is stored by two memristors.

**FIGURE 5:**
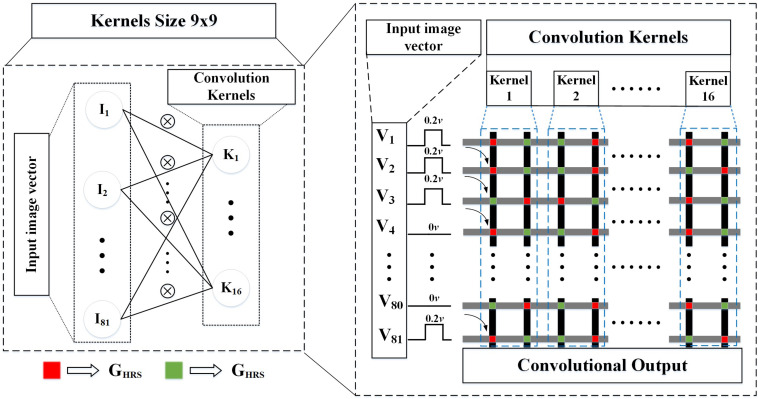
Memristor array structure of the convolution kernel. The pixels in the sliding window are convolved with 9 × 9 kernel using the memristor array.

A demonstration about how to perform a convolution operator in memristor crossbar is given in Figure 4. The input image with two dimensions would be reshaped into one dimension, and the binarized pixels are directly converted to DC or impulse voltages to input the memristor array. And then the detail methods of mapping the 16 convolution kernels in the CFB-CNN architecture to memristor array is illustrated in Figure 5.

**FIGURE 6:**
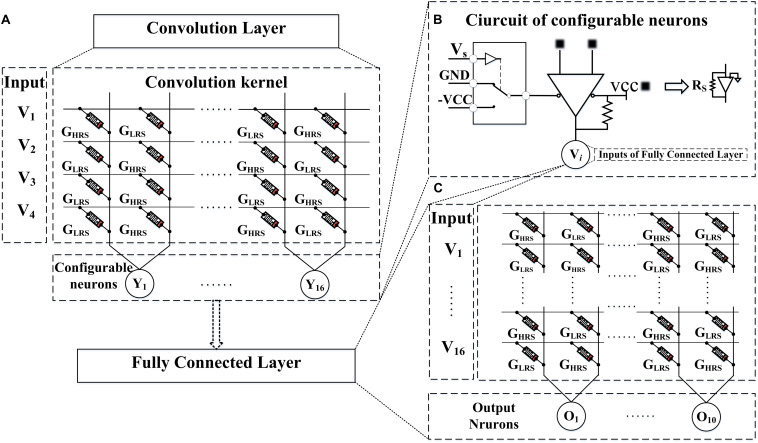
The example depicts the hardware implementation of the CFB-CNN architecture, **(A)** module of convolution layer, **(B)** detailed circuit of configurable neurons, **(C)** module of fully connected layer.

One convolution operation, as shown in Figure 6, is taken as an example to illustrate the hardware architecture of the entire network. In this example, the input image and kernel size are both 2 × 2, and the number of the convolution kernels is 16. And the configurable neurons with two types of activation functions, following behind the convolution layer, are implemented by the circuit in Figure 6B. This circuit mainly consists of one Single-Pole-Double-Throw switch (SPDTs) and one comparator. The function of the SPDTs is to decide which types of output value, ±1 (±VCC) or 0,1 (GND,VCC) the comparator will be. To be more specific, the input of SPDTs *V*_*s*_ would be set to be VCC if a neuron with the types of value 0,1 (GND, VCC) were expected. Otherwise, it would be set to be GND. The current values obtained by the VMM operation are converted to voltages by op-amp, afterward these voltages are input to the comparator. And the output of the comparator is the input of the fully connected layer.

Here, the convolution kernel size of the CFB-CNN architecture is set to be 9 × 9, and the number of the kernel is 16. As shown in Figure 5, each kernel is arranged in two columns, therefore, each one will consume 9 × 9 × 2 memristors. And all the sixteen kernels need to execute 400 convolution operations. Hence, the total number of memristors that the convolution layer required is 162 × 400 × 16. As mentioned above, sixteen feature maps with size being 20 × 20 would be output by convolutional layer, as a result of which, the number of neurons input to fully connected layers is 20 × 20 × 16. And the number of weights in fully connected layer is 20 × 20 × 16 × 10, on account of the number of categories needed to be classified of recognition task. Consequently, 20 × 20 × 16 × 10 × 2 memristors are required in fully connected layer.

## Simulation and Experimental Results

### Simulation About Network Parameters Selection

**FIGURE 7:**
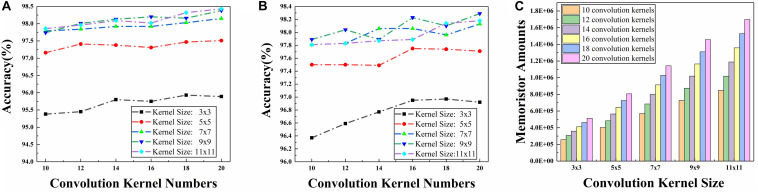
Simulation results about convolution kernel size and amounts impact on network performance and number of memristors that required in hardware implementation of the CFB-CNN architecture, **(A)** simulation results for ±1 MD, **(B)** simulations results 01 MD, **(C)** memristor amounts required in hardware implementation of the CFB-CNN architecture.

Since there is no pooling layer in the CFB-CNN architecture, the size of the feature maps which poses a significant impact on network performance is only determined by the size of the convolution kernel. Beyond that, the input number of the fully connected layer also depends on kernel size. And the number of memristors in fully connected layer is also determined by the number of neurons input to it. Therefore, network performance and the total number of memristors required are seriously affected by the kernel size. And the results of simulation about the influence of convolution kernel size and kernel numbers on network performance and the total number of memristors required are given in Figure 7.

As shown in Figure 7C, for the problem of MNIST dataset identification, whether the neuron mode is configured to be ±1 or 0,1, the memristor amounts soars with the increase of the convolution kernel size ranging from 3 × 3 to 11 × 11. As for the recognition performance of the CFB-CNN architecture, it improves continuously when the kernel size increases from 3 × 3 to 9 × 9 as shown in Figures 7A,B. However, when the convolution kernel size is set to be 11 × 11, there is no evident improvement in network performance except for the increased hardware consumption. Therefore, in order to make the network have a higher recognition rate and less hardware consumption, the kernel size is configured to be 9 × 9. Another information acquired, under the condition where the convolution kernel size is configured to be 9 × 9, is that when the number of kernels is set to be 16, 18, and 20, respectively, the CFB-CNN architecture can perform well with recognition rate more than 98% no matter what kind of the hidden neuron mode is configured. However, the memristor amounts soars up with the increase of kernel number as shown in Figure 7C, making it difficult for hardware implementation of the CFB-CNN architecture due to the enormous overhead of memristors. Therefore, the size and number of convolution kernels in the CFB-CNN architecture is configured to be 9 × 9 and 16.

### Effect of Memristance Fluctuation and Array Yield on Network Performance

Firstly, the weights in the well-trained CFB-CNN architecture are programmed to the memristor array using the method in Figure 4. Then, the memristor based CFB-CNN architecture is tested on the MNIST dataset. Without considering any non-ideal characteristics of memristors, the CFB-CNN architecture based on memristor array has a recognition rate of 98.2% for binary input images and 98.37% (4-bit-±1 MD) or 97.73% (4-bit-01 MD) for 4-bit input images. Although the manufacturing technologies of binary devices are relatively mature, the factors that affect network performance, such as yield and device resistance fluctuations, cannot be ignored. Hence, the Monte Carlo method is used to analyze the influence of memristance variation and yield of array on network performance. In simulation, the memristance of HRS and LRS corresponds to 1 MΩ and 1 kΩ, respectively (Liu et al., 2016). The excitation signal adopts a voltage pulse with a fixed amplitude of 0.1 V, and the pulse width is 10 ns. That is to say, when the pixel value of the input image is binary, viz., 0 and 1, the pixel value “1” will be encoded as one pulses, in contrast, the pixel value “0” means that there is no pulse. Likewise, when the input of the image is 4 bits, the memristor based CFB-CNN architecture inputs are distributed from 0 to 15 pulses, to be more specific, when the pixel value is 8, it will be encoded as 8 pulses with amplitude and pulse width being 0.1 V and 10 ns. For the defect of device variation, it is assumed that the memristance variation follows normal distribution, which can be described as Eq. 5.

(5) $$R∼N\left(\mu_{0},\sigma^{2}\right)$$

Where μ_0_ represents the average values, viz., 1 MΩ in HRS and 1 kΩ in LRS. The parameter σ should satisfy the equation that depicts as Eq. 6.

(6) $$\sigma =\mu_{0}\times \text{rate}$$

where the parameter rate represents the percentage of variation varying from 0 to 30%.

To analyze the influence of device variation on the CFB-CNN architecture, the memristors suffered from the defect of variation should be mapped back to the logical weights in CFB-CNN. Specifically, suppose that the two memristors corresponding to weight “1” experience a resistance fluctuation, their conductance will change from G_LRS_ and G_HRS_ to G’_LRS_ and G’_HRS_, where G’_LRS_ and G’_HRS_ in memristor array are the actual representation of the weight “1,” while G_LRS_ and G_HRS_ in memristor array are the ideal representation of the weight “1.” Consequently, this two memristors suffering from the fluctuation no longer correspond to a perfect “1” weight in the CFB-CNN architecture. And the actual weight in CFB-CNN corresponding to the two memristors after experiencing the fluctuation should be obtained from Eq. 7.

(7) $$w_{i}^{r}=x=\frac{1}{k}y_{r}=\frac{1}{G_{LRS}-G_{HRS}}y_{r}$$

where *y*_*r*_ represents the difference of the conductance of the two memristors after experiencing a fluctuation in the memristance, and $w_{i}^{r}$ represents the actual weight in CFB-CNN corresponding to the two memristor after experiencing variation.

For the yield problem, an assumption has been made that when the device in array is damaged, it means that this memristor sticks at G_HRS_ (S-A-H) or G_LRS_ (S-A-L). During the process of simulation, the resistance in memristor array is randomly changed to be G_HRS_ or G_LRS_ for emulating the defect of S-A-H or S-A-L (Liu et al., 2017). And the damaged device has a 50% chance of being stuck at G_HRS_ or G_LRS_ when there exists a yield problem.

**FIGURE 8:**
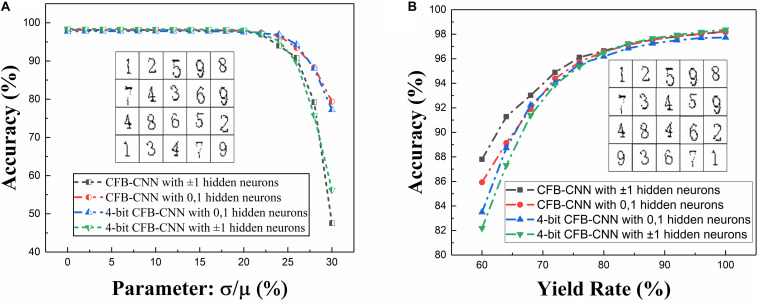
Impacts caused by different types of defects on 01 MD and 4-bit-01 MD or ±1 MD and 4-bit ±1 MD, **(A)** recognition rate of CFB-CNN architecture on MNIST dataset with memristance variation varying from 0 to 30%, **(B)** recognition rate of CFB-CNN architecture on MNIST dataset when damaged device rate is varying from 0 to 40%.

The impact of device variation on network performance when neurons are configured to be different types is firstly analyzed. Figure 8A shows the impact on network performance when device variation ranges from 0 to 30%. Obviously, when the memristance variation is 26%, the recognition rates of the model of 01 MD and 4-bit-01 MD are 93.43 and 94.3%, respectively. As for the model of ±1 MD and 4-bit-±1 MD, they degrade to about 90.85 and 89.68% in separate. And when the memristance variation is 30%, the recognition rates of ±1 MD and 4-bit-±1 MD are declined to 47.53 and 56.19% separately, but the model of 01 MD and 4-bit-01 MD still achieve about 79.38 and 77.2% recognition rates severally.

**FIGURE 9:**
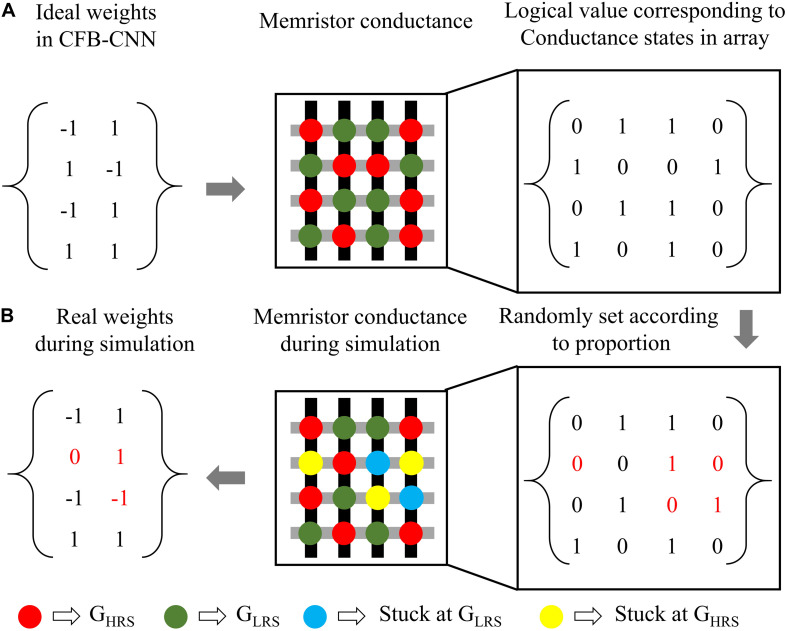
The illustration of the simulation process about yield problem, **(A)** relationship between the weights in CFB-CNN architecture and conductance states in memristor array, **(B)** description about how to simulate the damaged device in Python.

After that, the impact of yield rate in memristor array on the CFB-CNN architecture’s performance is also analyzed on MNIST dataset. And, to facilitate the analysis of impact of yield rate in array on the CFB-CNN architecture, the logical value 0 and +1 are used to represent the low conductance state (G_HRS_) and the high conductance state (G_LRS_) in memristor array, respectively, so the relationship between the weights in the CFB-CNN architecture and conductance states in memristor array is similar as in Figure 9A. Thereby, in the process of simulating the influence of the array yield on network performance, the original weight matrix containing only +1, −1 should be expanded into a weight matrix containing only 0, 1 according to the relation shown in Figure 9A. Subsequently, the values in the matrix are assigned “0” (G_HRS_) and “1” (G_LRS_) according to the ratio of 0∼40%, as shown in Figure 9B. As shown in Figure 8B, with the yield rate being 64%, the recognition rate of the ±1 MD remains about 91.28%. While the recognition rate of 01 MD drops nearly to 89.13%. However, the recognition rates of 4-bit-01 MD and 4-bit-±1 MD models both decrease slightly, and their recognition rates are 88.73 and 87.32%, respectively.

### Impact of Neuron Output Variation on Network Performance

**FIGURE 10:**
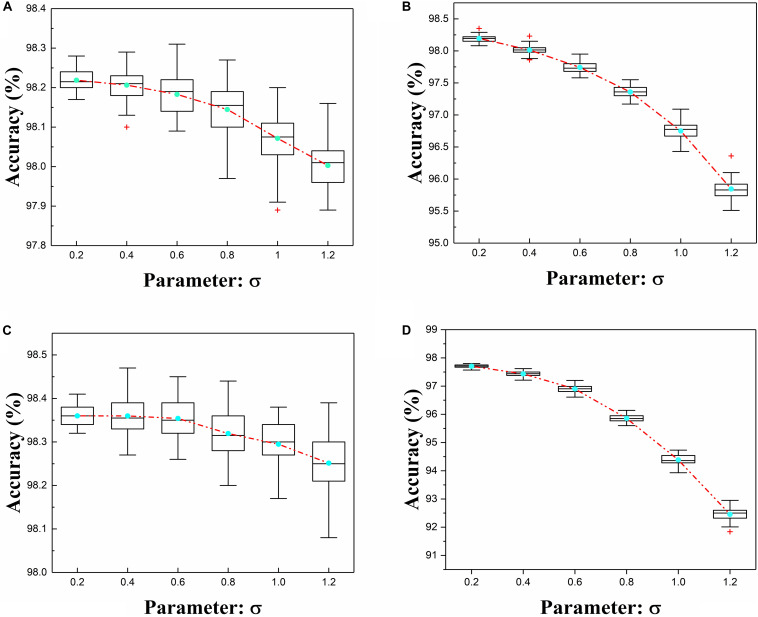
Neuron output variation analysis results of ±1 MD (left, **A**) and 4-bit-±1 MD (bottom left, **C**) or 01 MD (right, **B**) and 4-bit-01 MD (bottom right, **D**), and each box corresponds to 50 experiment results.

In addition to the defect that caused by device, the noise in the circuit can also lead to a fluctuation on the neuron outputs, which may also degrade the CFB-CNN architecture performance. Hence, it makes sense to investigate the influence of neuron output fluctuation on performance of this proposed architecture. The configurable neurons would output impulse voltage with amplitude to be 0 V (GND), 3.3 V (VCC), or ±3.3 V (±VCC), which corresponds to the logical value 0, 1, or ±1 of the configurable neuron output in the trained CFB-CNN architecture, respectively. Here, an assumption has been made that the fluctuation of neuron output value satisfies the relation of normal distribution, and the expectations of the distribution are 0 V, 3.3 V, or ±3.3 V, respectively. That means, if the configurable neuron needs to output an ideal voltage 0 V, its real output voltage would be determined by the relation *N*∼(0,σ^2^). In the same way, the output value after experiencing variation should also be mapped back to neuron output in the CFB-CNN architecture. When the output value experiencing fluctuation changes from 3.3 to 3.63 V, the neuron output in CFB-CNN would change from 1 to 1.1. The parameter σ, ranging from 0 to 1.2, reflects the range of the fluctuation. The analysis results of neuron output fluctuation as shown in Figure 10.

Obviously, when the neuron is configured as ±1, the fluctuation of the neuron has less influence on the network performance no matter what kind of input the CNN-CFB architecture is, because it is more difficult to achieve the transition from the logic level “−1” to the logic level “+1” than the logic level “0” to the logic level “1.”

### Simulation of CFB-CNN With Pooling Layer

In this section, a controlled experiment, comparing the CFB-CNN architecture with pooling layer (MODEL1) with the counterpart without pooling layer (MODEL2) from the perspective of defects tolerance and area consumption, is given to demonstrate why the CFB-CNN architecture does not need a pooling layer. And the comparison results about networks’ robustness under the condition where the yield rate and device variation are ranging from 0 to 40% and 0 to 30% in separate. However, when the mode of the hidden neurons in MODEL1 is configured to be 0,1, the condition of the analysis for neuron output variation is ranging from 0 to 3% being different from that when the hidden neurons are configured to be +1 or −1. And in order to exclude the influence of other factors, following limitations are made:

• Firstly, in order to eliminate the difference on the robustness of the network on non-ideal devices caused by different convolution kernels and the size of feature maps that input to fully connected layer, a large convolution kernel size, namely 7 × 7, is also used in MODEL1.
• Secondly, to obtain the feature map size of 20 × 20, the pooling layer kernels are set to be 3 × 3 and the step size is 2.
• Finally, in order to avoid the different performance resulting from difference in the number of memristors used in MODEL1, the number of convolution kernels is 21. In this case, the number of memristors used in MODEL1 is roughly the same as the number of memristors used in MODEL2.

**FIGURE 11:**
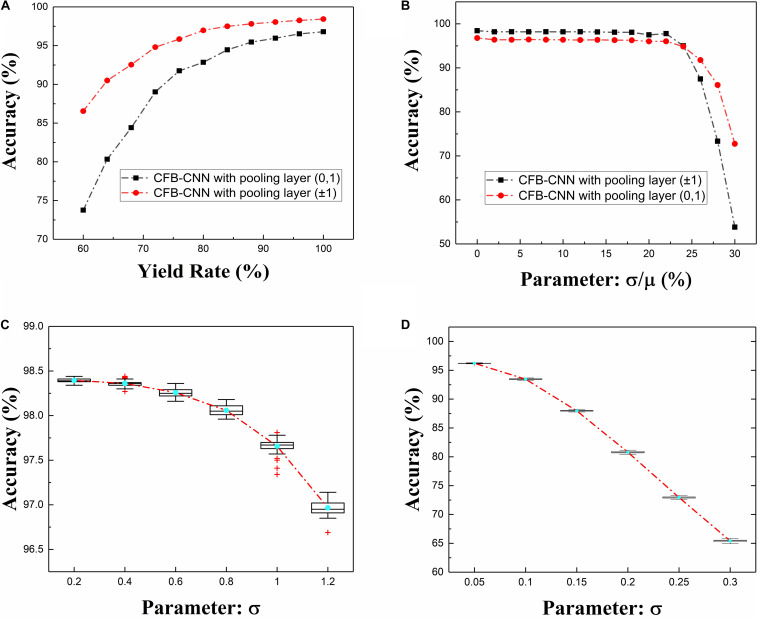
The influence of device non-ideality on the recognition rate of the CFB-CNN architecture with pooling layer, **(A)** the influence of array yield on the recognition rate of the CFB-CNN architecture, **(B)** the influence of the memristance variation on the performance of the CFB-CNN architecture, **(C,D)** the influence of the neuron output variation on the recognition rate of the CFB-CNN architecture with pooling layer and hidden layer neurons configured as ±1 or 0, 1, respectively.

The ideal recognition rate of the MODEL1 is 98.43 and 96.79% corresponding to the neuron mode of +1 or −1 and 0,1, respectively. Under the condition where the neuron mode is configured to be +1 or −1, its performance has a certain improvement compared with MODEL2. However, when the neuron mode is configured to be 0,1, the recognition rate of the MODEL1 is 1.43% lower than MODEL2. As shown in Figures 10, 11A,B, when the hidden layer neuron configuration of the MODEL1 is 0,1, the robustness of MODEL1 to device non-ideality is declined slightly. What’s more, the neuron output variation degrades the performance of the CFB-CNN architecture sharply as shown in Figures 11C,D. Last but not least, the complexity of hardware implementation for MODEL2 declines significantly compared with MODEL1 resulting from the fact that no pooling layer is needed in MODEL2. Based on the above analysis, the CFB-CNN architecture without pooling layer cannot only simplify the design of the circuit, but also has excellent robustness to neuron output variation at the cost of negligible recognition loss. Consequently, the CFB-CNN architecture without pooling layer is adopted in this work.

### Simulation With Different Proportions of Neuron Configuration

As mentioned in Section “Convolutional Neural Network,” the proportion μ could be adjusted to determine how many feature maps to be binarized to +1 or −1. And in Sections “Effect of Memristance Fluctuation and Array Yield on Network Performance” and “Impact of Neuron Output Variation on Network Performance,” we just present the non-ideal analysis results of the memristor based CFB-CNN architecture with μ being 0 or 1. For a more detailed analysis of the architecture, a simulation with different proportions of neuron configuration is given. Table 1 shows the recognition performance of the CFB-CNN architecture on MNIST dataset without considering defects.

**TABLE 1:** Recognition rate on MNIST varying with proportion.

Proportion (μ)	1	0.875	0.75	0.625	0.5	0.375	0.25	0.125	0
Recognition rate (%)	98.2	98.17	98.17	98.01	98.15	98.18	97.97	98.08	98.22

**FIGURE 12:**
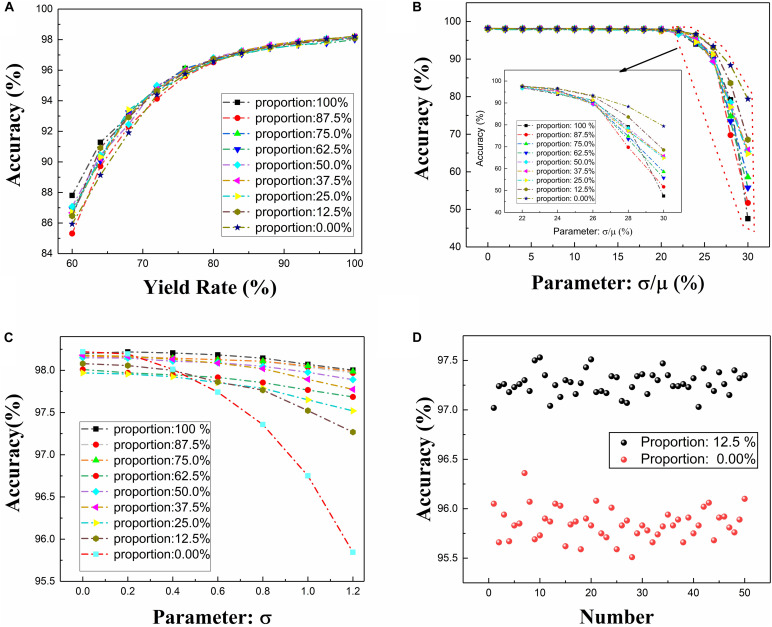
The CFB-CNN architecture with different proportions of neuron configuration with different types of defects existing in memristor array, **(A)** the impact of the yield ranging from 60 to 100% in memristor array on the CFB-CNN architecture, **(B)** the impact of memristance variation on the performance of CFB-CNN architecture, **(C)** the impact of neuron output variation on the CFB-CNN architecture, **(D)** detailed distribution of test accuracy with the parameter σ being 1.2 and the proportion of the neuron configuration being 12.5 and 0.0%, respectively.

As shown in Table 1, the network gets good recognition performance under different configuration. Similarity, considering non-ideal conditions mentioned above, the performance of this architecture with different proportions of neuron configuration is analyzed and shown in Figure 12.

As shown in Figures 12A,B, the model of ±1 MD shows excellent adaptability to the yield defects. And the architecture of 01 MD has good adaptability to the memristance variation defects. As shown in Figures 12C,D, the robustness of the CFB-CNN architecture to the neuron output variation with the proportion of the neuron configuration being 0% is improved by utilizing two kinds of activation functions. Moreover, with the different proportions, the robustness of the network to different device defects is also varied. Hence, we can choose the appropriate proportion to improve the performance of the memristor based CFB-CNN architecture according to the characteristic of the memristor array.

### Implementation Overheads

To evaluate the hardware overheads of the memristor based CFB-CNN architecture, the size of Pt/HfO_2_:Cu/Cu memristor (Liu et al., 2016) ranging from 2 to 5 μm is used for analyzing. And the impulse voltages with amplitude and width being 0.2 V and 50 ns are used as the read and programming conditions, while the voltages with (1.5 V/50 ns) and (−2 V/50 ns) are used for setting and resetting operation, respectively. The evaluation for energy consumption of the CFB-CNN architecture consists in energy consumption in convolutional layer and fully connected layer. And for the inference of one picture in MNIST dataset, an estimation method similar to ref (Li et al., 2017) is used to evaluate the energy consumed in the fully connected layer, while the energy consumed in convolutional layer is estimated as follows:

• Firstly, we should count how many points with a pixel value of “1” are included in the test set after binarization processing, and then calculate the average number of pixels included in each image. According to statistics, the total number of pixels is around 1.52e6, and so the average number of pixels contained in each image is 152. That is to say, each image will contain 152 pulses for input.
• Secondly, a geometric probability model is introduced to approximate the number of pixels included in each sliding process of the convolution kernels, since the pixels included in the convolution kernels during each sliding process are uncertain. Specifically, when the size of the convolution kernel is 9 × 9, it would account for the percentage of 81/784 in a 28 × 28 image. Therefore, the number of pixels contained in the convolution kernel in each sliding process is 152 × 81/784, approximately 16. Which means that, there would be 16 rows of valid inputs in the memristor array as shown in Figure 5. Hence, in each convolution process, 16 × 32 memristors will participate in the calculation, and the number of devices with low resistance state and high resistance account for half, respectively.
• Finally, the energy consumption of one convolution operation is calculated from Eq. 8. And the total energy consumption of the convolutional layer is 400 times of that consumed by one convolution operation.

(8) $$E_{f}=\frac{V_{R}^{2}}{R_{ON}}\times N_{ON}\times \Delta t+\frac{V_{R}^{2}}{R_{OFF}}\times N_{OFF}\times \Delta t$$

where *V*_*R*_ represents the input voltage (Here assumed to be 0.1 V with pulse width being 50 ns), *R*_*ON*_ and *R*_*OFF*_ represent the memristor state in LRS and HRS in separate, while *N*_*ON*_ and *N*_*OFF*_ represent the number of memristors in LRS and HRS, respectively.

In the same way, for the model of 4-bit-01 MD or 4-bit-±1 MD, the method of averaging is used to calculate the number of pulses that will be included in each image when the input images are compressed into 4 bits. To be more specific, the sum of the pixels of the test set image quantized to 4 bits is first calculated, and then it is converted into the corresponding number of pulses according to the coding rules. And finally the average number of pulses contained in each image is obtained, that is 2245. Since, there are 28 × 28 pixels in an image, we assume that each pixel is able to be encoded into 2245/784, about three pulses. In addition, since the size of the convolution kernel size is 9 × 9, there will be 9 × 9 rows of inputs in the memristor array. Hence, in each convolution process, 81 × 32 memristors will participate in the convolution calculation, and the high and low resistance memristors account for half in separate. Terminally, the energy consumption of one convolution operation for the model of 4-bit-01 MD or 4-bit-±1 MD is calculated as Eq. 9.

(9) $$E_{4bit}=3E_{f}$$

As shown in Table 2, the energy cost is different between the CFB-CNN architecture at different modes. This is mainly because when the hidden layer neuron is configured to be 0,1, part of the voltage pulse amplitude input to the fully connected layer is 0 V, therefore, some memristors in the array are not involved in the convolutional operation. Table 3 shows the comparison of the performance between our network and other similar designs. We note that the proposed CFB-CNN architecture offers comparable performance.

**TABLE 2:** Implementation overheads.

	Input Type	Latency	Area (F^2^)	Energy (J)	Device numbers
CFB-CNN (0,1)	0,1	50 ns	4.6592e6	5.4270e-8	1.1648e6
CFB-CNN (±1)	0,1	50 ns	4.6592e6	8.3282e-8	
CFB-CNN (0,1)	4 bits	50 ns	4.6592e6	7.8021e-7	1.1648e6
CFB-CNN (±1)	4 bits	50 ns	4.6592e6	8.1041e-7	

**TABLE 3:** Performance comparison with other publications.

Parameters	Reference (Yakopcic et al., 2017)	Reference (Sun et al., 2019a)	This work	
Network Structure	6c-2s-72c-2s-FCL	20c-2s-FCL	16c-FCL	16c-FCL
Memristor Amount	7,898,026	1,336,000	1,164,800	1,164,800
Activation Function	Sigmoid	Abs	±1	0,1
Accuracy	98.92%	99%	98.2%	98.22%
Power Dissipation (W)	>10	0.156–3.142	1.666	1.085

We can notice that the number of memristors required in the CFB-CNN architecture is the least, in the case of that, it can still achieve the similar recognition rates compared with other two works. And the power dissipation of the CFB-CNN architecture is also more competitive compared with other two advanced works, which means that the proposed CFB-CNN architecture is suitable for embedded implementation.

## Conclusion

In this work, we propose a CFB-CNN architecture, whose inputs, weights, and neurons are all binary values. In addition, we have given the scheme about hardware implementation of configurable neurons. Furthermore, the CFB-CNN architecture is verified to be SNN-compatible by encoding the pixel value of the input image as number of spikes for simulation, which means that both SNNs and CNNs are application candidates for this architecture. Certain simulations have been conducted on the MNIST to verify the CFB-CNN architecture. The results show that when the architecture uses a two-layer network, it has a good classification performance on the MNIST data set, with a recognition rate of 98.2%. In addition, this architecture shows excellent robustness to device non-ideal characteristics, specifically, when the yield rate is about 64% and the hidden neuron mode is configured as −1 and +1, the CFB-CNN architecture can achieve about 91.28% recognition accuracy. Whereas the resistance variation is about 26% and the hidden neuron mode configuration is 0 and +1, the CFB-CNN architecture can obtain about 93.43% recognition accuracy. To sum up, the network architecture of this work is based on binary memristive synaptic device, and it has better robustness to the defect of device which means that it is suitable for embedded application of memristors.

## Data Availability Statement

Publicly available edatasets were analyzed in this study. This data can be found here: http://yann.lecun.com/exdb/mnist/index.html.

## Author Contributions

LH and HL designed the architecture of full-binary CNN, the circuit of configurable neurons and the experiments. WW and ZL gave help for designing methods of simulation. LH performed the simulation work and conducted the experiments. LH, HL, ZL, WW, HN, and QL contributed to the writing and editing of the manuscript. JD supervised the project. All authors discussed the results.

## Conflict of Interest

The authors declare that the research was conducted in the absence of any commercial or financial relationships that could be construed as a potential conflict of interest.
